# Analysis of Attitudes About COVID-19 Contact Tracing and Public Health Guidelines Among Undocumented Immigrants in the US

**DOI:** 10.1001/jamanetworkopen.2021.37719

**Published:** 2021-12-08

**Authors:** Hye Young Choi, May Sudhinaraset

**Affiliations:** 1 Department of Social and Behavioral Sciences, Harvard T.H. Chan School of Public Health, Boston, Massachusetts; 2 Department of Community Health Sciences, Fielding School of Public Health, University of California, Los Angeles, Los Angeles

## Abstract

This qualitative study examines the factors associated with undocumented immigrants’ ability and willingness to follow COVID-19 contact tracing and public health guidelines.

## Introduction

More than 10.7 million undocumented immigrants and 8 million citizens with at least 1 undocumented family member live in the US.^1^ Evidence shows that immigrants are at increased risk for COVID-19 infection^2,3,4^ and have high levels of distrust in public systems.^5^ Contact tracing is an effective way to mitigate disease transmission but requires trust and cooperation among infected persons and their contacts. Using the Centers for Disease Control and Prevention sample contact tracing script as a framework, we describe undocumented immigrants’ attitudes about contact tracing and challenges that may be a factor in their ability to follow contact tracing and public health guidelines.

## Methods

This qualitative study used data from 20 in-depth interviews conducted between June and October 2020 to examine the social, economic, and health implications of COVID-19 for undocumented immigrants. We used snowball sampling to recruit a purposive sample of 20 undocumented immigrants representing equal proportions of male and female Asian and Latinx people, who self-reported their race and ethnicity. All participants provided verbal informed consent and were virtually interviewed using a semistructured interview guide codeveloped by a community advisory board that included undocumented immigrants and subject matter experts. Specific questions regarding contact tracing were integrated after the fourth interview based on community reports about challenges engaging immigrants in contact tracing. To assess potential differences in attitudes based on inclusion in the Deferred Action for Childhood Arrivals (DACA) program—a federal program that provides temporary work authorization and protection from deportation to eligible undocumented immigrants—we ascertained participants’ DACA status by asking, “Are you currently a DACA recipient?” Using grounded theory, 1 of us (H.Y.C.) and a research assistant developed a codebook and analyzed all transcripts between October 2020 and February 2021. Subheadings in the Centers for Disease Control and Prevention’s Contact Tracer’s Interview Tool: Notifying People About an Exposure to COVID-19^6^ were used as larger domains to organize participant quotes. This study was approved by the University of California, Los Angeles Institutional Review Board and follows the Standards for Reporting Qualitative Research (SRQR) reporting guideline.

## Results

Of 20 participants (mean [SD] age, 27 [3.7] years; 10 men and 10 women) included in the sample, 16 (80.0%) were asked about their familiarity with contact tracing, and 6 (37.5%) had never heard of contact tracing before the interview. Among 10 participants (62.5%) with some awareness of the process, 2 (20%) reported ever speaking with a contact tracer. After learning about the principles of contact tracing, including privacy and confidentiality as well as select contact tracing prompts (ie, home address, work address, names of cohabitants), 13 of 20 participants (65.0%) worried about disclosing information, and of those, 10 (76.9%) specifically cited immigration-related concerns.

We found that numerous questions in the Centers for Disease Control and Prevention’s sample contact tracing script have the potential to elicit fear, discomfort, inaccuracies, or nonresponse among undocumented immigrants based on participant concerns about breach of information, protecting undocumented parents from deportation and language discrimination, and distrust in government agencies (Table 1). For example, both Asian and Latinx participants doubted the quality of language access that could be offered to their parents and said their parents’ lack of DACA status, and thus greater vulnerability to deportation, made them reluctant to share information on their parents’ behalf.

**Table 1.** Participant Responses to Questions From the Centers for Disease Control and Prevention Sample Contact Tracing Script

Contact tracing script^a^	Participant response	Reason for concern
**Introduction**		
Who is their/your parent/guardian? How can I reach their/your parent/guardian?	“I guess the main fear is, because I have DACA, at least in terms of ICE, it’s much easier for them to go after my parents than it is to go after me or someone in my situation.” —*Asian participant in the DACA program*	Parental lack of DACA and vulnerability to deportation
What language(s) do you feel most comfortable speaking?	“I don’t want [my parents] to be put in situations where they would have to answer questions. And since they can’t speak English—and I’m pretty sure there will still be someone from the government who knows how to speak Korean—but on the off chance that there isn’t, and they have to answer for themselves in English or broken English/Korean, I wouldn’t want them to be in that situation.” *—Asian participant in the DACA program*	Parental lack of DACA and vulnerability to deportation, potential for language discrimination
	“So, always, language barrier is [why I am reluctant to share] because I’ve had experiences where prior to COVID, I would go with my parents to like, you know, the hospital or whatnot. And I just had to be there because if I wasn’t, they wouldn’t get the medical care that they deserve. And so that’s always been a concern for me.” *—Latinx participant in the DACA program*	Potential for language discrimination
**Locating and contact information**		
Where do you stay (or live)? What is your address?	“Just knowing ICE works closely with the Trump administration and how they’re doing everything in their power…to ‘get rid of us,’ that they don’t want any undocumented person here.” *—Latinx participant in the DACA program*	Distrust in government agencies
**Work**		
Where do you work (name, location(s), hours)?	“[My mom] doesn’t have DACA or any form of legal help, so she works under the table.…When she tells people [where] she works, she’s like, ‘Oh, I work at home.’ It’s like this whole maneuvering.” *—Latinx participant in the DACA program*	Parental lack of DACA and vulnerability to deportation
What other things do you do to earn money besides the job you just described?	“Well, it’s none of your business. And I’m trying to hustle over here.…You don’t give me a social security number? Well, shoot. I gotta hustle somewhere else.…Sometimes you’re working under the table, there’s just all these different things that you just don’t report to the government.” *—Latinx participant not in the DACA program*	Distrust in government agencies
**Testing for COVID-19**		
Would you like to get testing for COVID-19 through your primary care provider or at the [insert local testing sites]? We have a list of test sites available.	“We’re also just kind of concerned about, like, if there is a need to present any paperwork…to be able to get COVID testing because, yeah, we understand that [Redacted] County has a different perception of immigrant families.” *—Asian participant not in the DACA program*	Distrust in government agencies
**Assessing living situation**		
What is your living situation? Who else lives with you? What are their names, ages, and relationships to you?	“I have DACA, and I have permission to just stay here and study and work. But my parents [not in the DACA program] are the ones who are living here too. So if [you ask], ‘How many people are living in your household,’ I’ll say, ‘I just live [by] myself.’” *—Latinx participant in the DACA program*	Parental lack of DACA and vulnerability to deportation

Abbreviations: DACA, Deferred Action for Childhood Arrivals; ICE, US Immigration and Customs Enforcement.

^a^ Excerpts from the Centers for Disease Control and Prevention sample contact tracing script.^6^

Participants also described a lack of resources to follow referrals or recommendations to access health care, quarantine, and negotiate work-based leave policies with employers. For example, when asked about informing employers about incident COVID-19 symptoms, participants mentioned they were denied sick leave or were immediately fired and felt financial pressure to keep working. We found considerable discordance between public health guidelines and undocumented immigrants’ retrospective or prospective abilities to follow the guidelines because of lack of access to immigrant-friendly health care, limited space to quarantine, low neighborhood assets, job insecurity, and lack of employee benefits and workplace protections (Table 2).

**Table 2.** Participant Responses to Public Health Guidelines Questions From the Centers for Disease Control and Prevention Sample Contact Tracing Script

Contact tracing script^a^	Participant response	Reason for concern
**Introduction**		
If you develop any of the symptoms we discussed earlier, you should reach out to your primary care provider or the health department liaison/public health nurse so that they can assist you in getting care.	“I don’t really have much memories of visiting a doctor or going into a doctor’s office. Mostly just because we really didn’t grow up with any medical insurance. Or in the times that—I guess it’s old school parents—it’s just, you don’t go to the doctor unless you’re like ‘dying’ dying.” *—Asian participant in the DACA program*	Lack of health insurance, low use of health services
From what you are describing, it sounds as though you should be seen by a health care provider to further evaluate your symptoms as soon as possible. You need to call 911 or go to the emergency department.	“We don’t have insurance. My mother does now, [through] emergency Medi-Cal. She found out about 2 months ago she had stage IV cancer, so we had to scramble [to find] insurance for her.…Around a week after my mom received her cancer diagnosis, [my sister] found out she was pregnant. She used to have insurance through [her] husband, but he was laid off because of COVID. And now she doesn’t have insurance so she’s also scrambling to find something.” *—Asian participant not in the DACA program*	Lack of health insurance
**Testing for COVID-19**		
It is important to go in for testing as soon as possible. When will you be able to go for testing?	“The closest [testing site] to us is about half an hour away.…If it was more accessible and more widespread in [Redacted] County, I would love to go personally, and I would definitely try to convince my family to go get tested at least like once.” *—Asian participant not in the DACA program*	Limited geographic access to services
**Assessing living situation**		
Would it be possible for you to have access to your own room and bathroom?	“They ran through questions with her, like, can you be isolated? Will you have your own restroom?…She basically said…that’s not possible; I don’t have my own wing of the house. But they basically told her, well, you can’t really stay here.…Maybe you can get a hotel room.” *—Latinx participant in the DACA program*	Limited space to quarantine
	“It’s a 2-bedroom apartment that we’re sharing between 5 people. So for the most part, I would just stay in my room, but…every now and then we would be in the same space.” *—Latinx participant in the DACA program*	Limited space to quarantine
**Assessing other supports**		
Do you have access to fresh water and enough food?	“Our water at the apartment isn’t really drinkable. And it’s more for purposes of washing.…There were a couple times that [bottled water] wasn’t at the store like they are now. And so, at the larger level, there was nothing that I could get.” *—Latinx participant in the DACA program*	Water scarcity, low neighborhood assets
	“I think the first day when my mom [told us] there was no food in stores, we had to ration everything and just eat really little of it. And I said, ‘OK, yeah, sure.’ Like…in college, we just ate little, like we just ate 1 time a day. It’s kind of normal for me.” *—Latinx participant in the DACA program*	Food insecurity, low neighborhood assets
How will you approach this discussion with your employer? Will you be getting paid sick leave from your employer?	“Because of my status, and the private contractor that I was working for wasn’t providing any health care benefits, all I could really do was just call the ER and just kind of explain to them what I was feeling and going through. And the best advice they gave me was to just stay home for 14 days. So, you know, I followed those orders, and I tried to send that message to my contractor. And, you know, so I decided to stay home for 2 weeks and that actually resulted in me not having that job anymore.” *—Latinx participant in the DACA program*	Lack of employee benefits and workplace protections
	“Even with finding out what the next step is and telling like my dad, ‘Hey, we’ve got to go to the hospital to find a social worker or a case manager to file a claim,’ he can’t. He refuses to take days off work, even though it’s him that has the health issues. Because if he stops working, especially in these times, [our financial situation] is going to get worse.” *—Asian participant in the DACA program*	Lack of employee benefits and workplace protections

Abbreviations: DACA, Deferred Action for Childhood Arrivals; ER, emergency room.

^a^ Excerpts from the Centers for Disease Control and Prevention sample contact tracing script.^6^

## Discussion

This qualitative study found that undocumented immigrants contend with unique challenges that may have implications for the ability of contact tracing programs to reach this population, including fears associated with policies that target undocumented immigrants (eg, immigration enforcement and eligibility rules for individuals in the DACA program and health coverage), language discrimination, and distrust of government agencies. These concerns were more salient among participants who indicated the need to protect their undocumented parents, revealing differential vulnerabilities and opportunities to engage immigrants in contact tracing.

The study is limited in terms of the generalizability of the findings because of the small, nonrepresentative snowball sample used. However, there is a critical lack of data among this population. We highlight important considerations for implementing contact tracing programs among undocumented immigrant populations.

Nationally, more than 16.7 million people in the US live with families with mixed immigration status or at least 1 family member with undocumented immigration status.^1^ Therefore, more research about the association between inclusive or restrictive immigration policies and the ability to follow public health strategies is needed to mitigate the health and social implications of COVID-19 for immigrant communities and beyond.
